# Factors Associated With *Helicobacter Pylori* Infection Among School-Aged Children From a High Prevalence Area in Vietnam

**DOI:** 10.3389/ijph.2023.1605908

**Published:** 2023-05-11

**Authors:** Thai Hoang Che, Tu Cam Nguyen, Vy Ngoc Thao Vu, Hiep Thanh Nguyen, Dung Thi Phuong Hoang, Xuan Minh Ngo, Dinh Quang Truong, Patrick Bontems, Annie Robert, Phuong Ngoc Van Nguyen

**Affiliations:** ^1^ Department of Biostatistics and Informatics, Faculty of Public Health, Pham Ngoc Thach University of Medicine, Ho Chi Minh City, Vietnam; ^2^ Pôle Epidémiologie et Biostatistique (EPID), Institut de Recherche Expérimentale et Clinique (IREC), Faculté de Santé Publique (FSP), Université Catholique de Louvain, Brussels, Belgium; ^3^ Department of Gastroenterology, City Children’s Hospital, Ho Chi Minh, Vietnam; ^4^ Faculty of Public Health, Pham Ngoc Thach University of Medicine, Ho Chi Minh City, Vietnam; ^5^ Department of Microbiology, Fundamental Sciences and Basic Medical Sciences, Pham Ngoc Thach University of Medicine, Ho Chi Minh City, Vietnam; ^6^ Faculty of Medicine, Pham Ngoc Thach University of Medicine, Ho Chi Minh City, Vietnam; ^7^ Department of Surgery, City Children’s Hospital, Ho Chi Minh City, Vietnam; ^8^ Gastroenterology, Hôpital Universitaire des Enfants Reine Fabiola, Université Libre de Bruxelles, Brussels, Belgium

**Keywords:** risk factors, *Helicobacter pylori*, Ho Chi Minh City, school-aged children, transmission route

## Abstract

**Objectives:** The study aimed to identify prevalence of *H. pylori* infection and associated risk factors among pupils of Ho Chi Minh city (HCMC).

**Methods:** A total of 1,476 pupils aged 6–15 years were enrolled in this cross-sectional study using multiple-stage sampling method. Infection status was assessed using stool antigen-test. A questionnaire was used to obtain socio-demographic, behavioral, and environmental factors. Logistic regression was performed to assess possible factors related to the infection.

**Results:** Of the 1,409 children included in the analysis, 49.2% were male and 95.8% were of Kinh ethnicity. About 43.5% of parents completed college or university. The overall prevalence of *H. pylori* was 87.7%. Infrequency of handwashing with soap after toilet, the use of only water to clean after toilet, crowded living areas, larger family size, and younger age were independently contributing to an increased prevalence of *H. pylori*.

**Conclusion:** *H. pylori* infection is highly prevalent in HCMC, and is associated with poor hygienic practices, crowded living areas, larger family size, and younger age. These findings highlight the importance of fecal-oral route and the attribution of crowded living conditions to the spreading of *H. pylori* in HCMC. Therefore, preventive programs should be set up with a focus on education of hygiene practices, and oriented to those living in crowded conditions.

## Introduction

*Helicobacter pylori* (*H. pylori*) was first discovered in 1983 by Warren and Marshall [1]. It is well-known as a pathogenic bacteria that causes gastric-related diseases such as gastritis and peptic ulcers [2, 3]. *H. pylori* is also recognized as bacterial carcinogen that contributes to the development of gastric cancer later in life [4]. Numerous studies have also reported a strong relationship between *H. pylori* infection and development of gastric cancer [3, 5, 6]. Furthermore, its role in extra-gastroduodenal disorders including iron deficiency anemia, and idiopathic thrombocytopenic purpura is also well documented in recent reports [3, 7]. These *H. pylori*-related gastric diseases and other-related disorders together cause over a million deaths each year, making *H. pylori* infection one of the most severe issues to public health worldwide.

*H. pylori* infection is mainly acquired during childhood and colonizes human gastric mucosa lifelong if not treated [3, 8]. School-going children are at high risk of infection and many infected pupils do not show any gastroduodenal symptoms up to adulthood [3, 9, 10]. According to a recent systematic review, the worldwide prevalence of *H. pylori* among children was found to be 32.3% [11]. It also reported that the incidence rate of *H. pylori* was significantly higher in low-income and middle-income countries than in high-income countries (43.2% vs. 21.7%) and in older children than in younger children (41.6% in 13–18 years old vs. 33.9% in 7–12 years old vs. 26.0% in 0–6 years old). Furthermore, several evidences indicated that *H. pylori* can be transmitted from person to person through possible routes, including oral-oral, and fecal-oral [3, 10]. Therefore, the bacteria can silently spread from asymptomatic infected individuals to others within family or in communities for an extended period of time, which might lead to high burden of *H. pylori* infection in community with a great impact on the public health system. However, the transmission patterns of *H. pylori* in children, compared to adults, remain scarce. Given that providing further insights on transmission modes and possible risk factors of *H. pylori* infection in asymptomatic children are essential for public health sector to build optimal prevention and reduction *H. pylori* programs.

*H. pylori* affects over 50% of the global population and its distribution dramatically varies both between and within countries [12]. The prevalence of *H. pylori* infection greatly decreased in developed countries, but it is still prevalent in developing countries—particularly in Asia with some countries reporting a prevalence of up to 90% [12]. Some recent studies pointed out that this different trend probably reflects the level of urbanization, sanitation, access to clean water, and varied socioeconomic status, but the exact reasons for this variation are not fully discovered yet [3, 12]. Like other developing countries in South-Eastern Asia, *H. pylori* infection in Vietnam remains high [12, 13]. A study conducted in Ha Noi city, the largest city in the North of VietNam, showed a sero-prevalence of *H. pylori* infection of 76.8% [14]. It was also reported that poor socioeconomic status, improper hygiene practices, and overcrowding living conditions were risk factors for getting *H. pylori* infection. A recent paper reporting on prevalence of *H. pylori* in school-aged children of Ho Chi Minh city (HCMC) showed the prevalence in the range of 80.2% in girls aged 12–15 years to 93.3% in boys aged 9–11 years [19]. There was no available community-based study focused on the mode of transmission and potential risk factors for acquiring *H. pylori* infection in HCMC, despite it is the biggest city in the South of VietNam. All previous studies on risk factors of *H. pylori* conducted in VietNam had small sample sizes and used serological tests which are known to have low accuracy in children [14–16]. In addition, there are big socio-economic and geographic disparities between HCMC and other investigated regions in VietNam. Therefore, identifying the route of transmission and associated risk factors for *H. pylori* infection in a large sample of school-aged children population of HCMC by using reliable tests are needed to develop preventive strategies in specific setting of HCMC.

We therefore conducted a community-based study of a large sample of school-aged children who were assessed for *H. pylori* infection by stool antigen test, to investigate possible risk factors and the route of transmission that may be associated with *H. pylori* infection in school-aged children in HCMC.

## Methods

### Study Design

We carried out a school-based cross-sectional study among public school pupils aged 6–15 years across 24 districts of HCMC in 2019. Situated in the Southeast region of VietNam, HCMC is the largest city with a population of 8.933.082 in total, of whom 1.5 million are pupils below 16 years of age [17, 18]. Like other parts of VietNam, there are two types of education systems in HCMC: a public system involving most of schools (*n* = 761, 96.7%) and a private system with few schools (*n* = 26, 3.3%) [17]. Out of the 761 public schools, there were 491 primary schools with grade 1st—5th (6–11 years) and 270 secondary schools with grade 6th—9th (12–15 years), representing a total of 1,077,105 pupils. Our study was carried out in public schools.

The sample size and the selection procedure have been reported previously [19]. Briefly, using the reported prevalence of 76.8% in HaNoi, at least 206 children per grade were required to reach a precision of 7.5% on 95% confidence interval for prevalence in population, assuming a loss rate of 20%, and adjusting for a design effect of 1.4. With 9 grades (1st—9th), the size of 1854 pupils or 9 pupils per class were needed to enroll in the study because there were 216 classes.

Pupils from public schools across 24 districts of HCMC were enrolled in the study by using a multiple-stage sampling method. In each of the 24 districts, one primary school and the closest secondary school were randomly selected. In each of these 48 schools, one class per grade was randomly chosen, and 9 children in the same class were randomly recruited into the study. A map that illustrated the number of school-aged children who participated in the study from each of 24 districts in HCMC was also included in the Supplementary Section.

### Eligibility Criteria

Asymptomatic healthy school-aged children of both sexes from 6 to 15 years old who studied at primary and secondary public schools in HCMC were invited to participate in the study. The criteria for eligibility included no documented previous *H. pylori* infection, no past history of gastroscopy or gastrectomy. Those who used antibiotics or bismuth-containing compound within last 28 days or used any proton pump inhibitors during past 14 days were also excluded from the study.

### Data Collection

A standard questionnaire was designed to obtain information regarding to socio-demographic characteristics, behavioral factors, lifestyle factors, and environmental factors of the children. A well-trained interviewer used the questionnaire to survey both pupils and their parents in the classroom at the end of the normal class. Two individual field data collectors independently entered the data obtained from questionnaire into Microsoft Excel. Data entry was validated by two other individual field data collectors and with paper version by an additional trained staff in case of disparities.

### Assess *H. pylori* Infection Status

A monoclonal enzyme-immunoassay (EIA) stool antigen test, Premier Platinum HpSA Plus test (manufactured by Meridian Bioscience, USA) was performed according to the manufacturer’s instructions and guidelines to determine *H. pylori* infection status [20]. Optical density (OD) values were used to categorize the status of *H. pylori* infection; OD values <0.100 were classified as negative and OD values ≥0.100 were classified as positive, as recommended by manufacturer [20]. Stool specimens with any water or urine were excluded from analysis.

### Statistical Analysis

Analyses were performed using Stata 17.0/IC software for Mac (TX: StataCorp LP). We report numbers with percentage for categorical variables and mean ± standard deviation for continuous variables. A household wealth index was defined as the first principal component analysis of 28 dummy variables coding for 28 household assets, as proposed by WHO for Demographic and Health Survey (DHS) in VietNam [21]. The wealth index was further categorized according to quintiles and reported on an ordinal scale. Associations between factors and feco-positivity were assessed using univariate logistic regression and reported as odds ratio with 95% confidence interval. Multivariable logistic regression analysis was performed to assess the independent contribution of each factor to *H.pylori* infection. Only factors associated with *H. pylori infection* with *p*-value <0.05 in the univariate analysis were considered in the multivariate regression. The statistical significance level was set to 0.05.

### Ethical Considerations

Written informed consent was obtained from both pupils and their parents. The purpose of the study, possible risks/benefits, and the rights were also explained to both pupils and their parents before obtaining the informed consent. The study protocol was approved by both the Ethical Review Committee and the Scientific Committee of the University of Medicine Pham Ngoc Thach and the Ethical Review Committee of Université catholique de Louvain—Brussels campus in Belgium. All data were anonymized and used for research purpose only.

## Results

**FIGURE 1:**
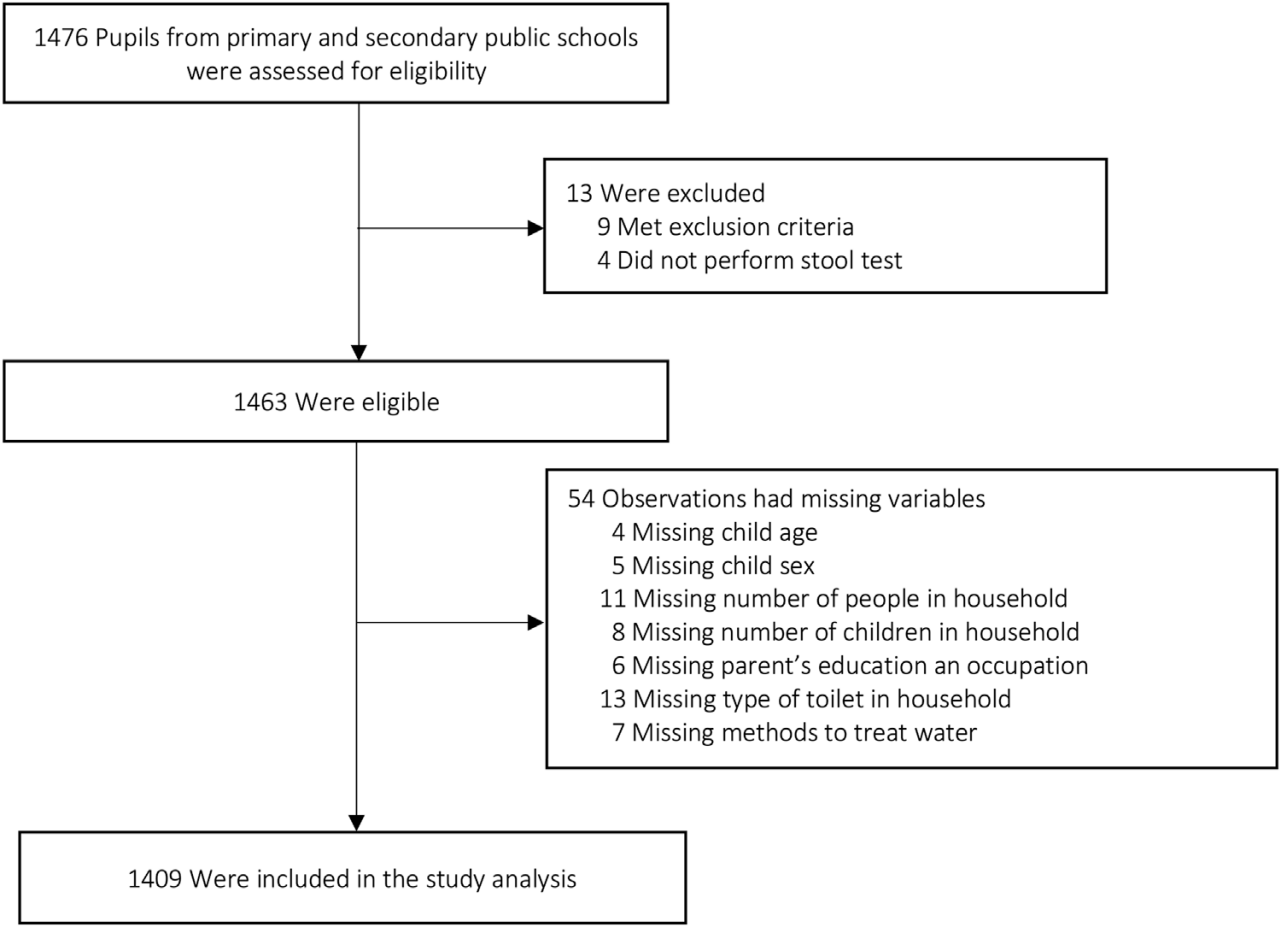
Flowchart of participants (Ho Chi Minh city, Vietnam. 2019).

A total of 1,476 school-aged children were enrolled in this study. Out of these pupils, 13 were excluded due to failure to meet inclusion criteria or not performing the stool test. Observations with missing child age, child sex, number of people, number of children in household, parent’s education, parent’s occupation, types of toilet, and methods to treat water were also excluded from the analysis. Ultimately, the remaining 1,409 pupils were included in the final analysis (Figure 1).

### Characteristics of Study Participants

Out of 1,409 participants, 693 (49.2%) were boys. The mean age of pupils was 10.1 ± 2.7 years with a range of 6–15 years. Almost participant’s ethnicity was Kinh (95.8%), while only 3.3% were Chinese. Nearly half of the parents had a high education level (college or university, 43.5%). Majority of parents worked in the factory (36.1%) or in the private business (29.2%). Most of families (68.1%) had 4–6 members and only one household (0.1%) had two members. The number of children in household was mainly two (61.9%) (Table 1).

**TABLE 1:** General characteristics of children investigated for *Helicobacter pylori* infection (Ho Chi Minh city, Vietnam. 2019).

Characteristics	Total
	n (%)
Ethnic group	
Kinh	1,350 (95.8)
Chinese	47 (3.3)
Others	12 (0.9)
Educational status of parents	
College/University	630 (43.5)
High school	133 (9.2)
Secondary school	512 (35.4)
Primary school	172 (11.9)
Parent’s occupation	
Factory worker	509 (36.1)
Office worker	277 (19.7)
Private business	411 (29.2)
Others	212 (15.1)
Wealth status of household^a^	
Poorest	263 (18.7)
Poorer	288 (20.4)
Middle	284 (20.1)
Richer	287 (20.4)
Richest	287 (20.4)
Family size	
≤2	1 (0.1)
3	145 (10.3)
4–6	960 (68.1)
>6	303 (21.5)
Number of children in household	
1	294 (20.8)
2	872 (61.9)
>2	243 (17.3)

^a^ Wealth index was defined as the first principal component analysis of 28 household assets, as proposed by World Health Organization for Demographic and Health Survey in Vietnam. It was categorized according to quintiles.

### Prevalence of *H. pylori* Infection

The overall prevalence of *H. pylori* infection was 87.7% (1,234/1,409). The prevalence was significantly higher in boys (89.5%, OR = 2.66, 95% Cl: 1.75–4.05) and in children aged 6–11 (89.4%, OR = 1.45, 95% Cl: 1.06–1.99). A trend of increasing prevalence was observed across four areas of HCMC, with rates of 80.6% in rural area, 87.6% in peri-urban area, 89.0% in urban area, and 91.6% in super-urban area.

### Association Between Possible Risk Factors and *H. pylori* Infection

Associations between demographic, socio-economic characteristics and *H. pylori* infection in univariate analysis are reported in Table 2. There was a strong inverse correlation between the educational level and *H. pylori* infection (*p* = 0.01), the prevalence of the infection was the highest (90.3%) for parents with lowest education (primary school). Having parents who completed primary school (OR = 2.05, 95% Cl: 1.28–3.28) was associated with a higher feco-positive rate. Parents who worked as factory workers (OR = 1.08, 95% Cl: 0.72–1.61) or office workers (OR = 1.17, 95% Cl: 0.72–1.89) were more likely to have a child infected with *H. pylori* than others (OR = 0.72, 95% Cl: 0.45–1.14), but these differences were not significant (*p* = 0.24). No associations were found with the ethnic group (*p* = 0.58) or with the wealth status of household (*p* = 0.63).

**TABLE 2:** Univariate association between *Helicobacter pylori* infection and demographic and socioeconomic factors (Ho Chi Minh city, Vietnam. 2019).

Factors	n/N^a^	%	OR (95% Cl)	*p*
Ethnic group				0.58
Kinh	1,181/1,350	87.5	1	
Others	53/59	89.8	1.26 (0.54–2.98)	
Educational status of parents				0.01
College/University	569/630	82.0	1	
High school	118/133	85.9	1.34 (0.85–2.13)	
Secondary school	440/512	88.7	1.73 (0.89–3.36)	
Primary school	141/172	90.3	2.05 (1.28–3.28)	
Parent’s occupation				0.24
Private business	360/411	87.6	1	
Factory worker	450/509	88.4	1.08 (0.72–1.61)	
Office worker	247/277	89.2	1.17 (0.72–1.89)	
Others	177/212	83.5	0.72 (0.45–1.14)	
Wealth status of household^b^				0.63
Poorest	223/263	84.8	0.80 (0.49–1.29)	
Poorer	256/88	88.9	1.15 (0.69–1.91)	
Middle	250/284	88.1	1.05 (0.64–1.74)	
Richer	254/287	88.5	1.10 (0.67–1.82)	
Richest	251/287	87.5	1	

^a^ n/N: number of infected children/number of children within sub-group.

^b^ Wealth index was defined as the first principal component analysis of 28 household assets, as proposed by World Health Organization for Demographic and Health Survey in Vietnam. It was categorized according to quintiles.

**FIGURE 2:**
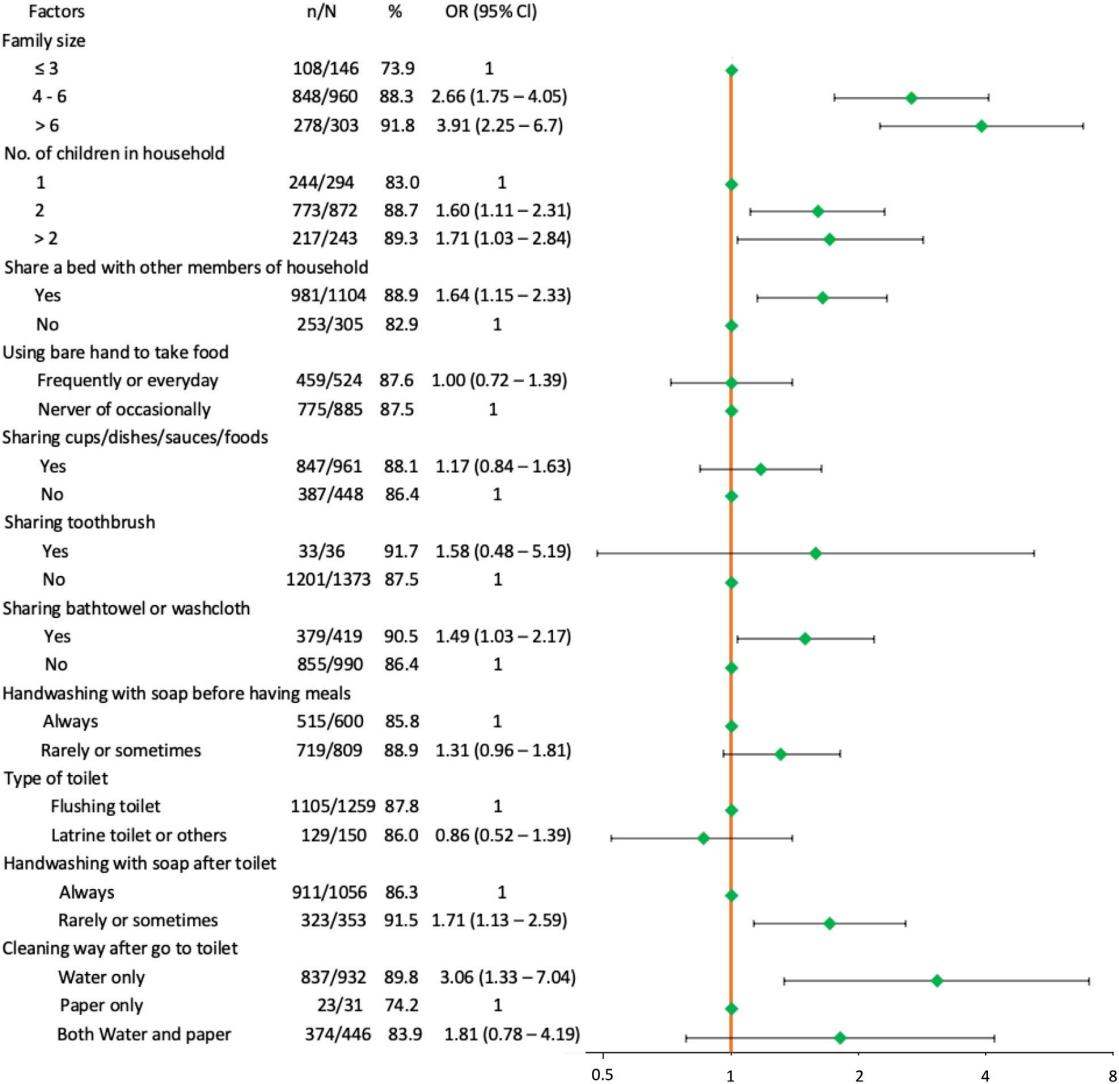
Forest plot of univariate association between *Helicobacter pylori* infection and crowding, lifestyle and hygiene factors. The orange solid vertical line is the reference line. Green dots are the estimated odds ratio. Horizontal black solid lines are 95% confidence intervals (Ho Chi Minh city, Vietnam. 2019).

Figure 2 presents relationship between *H. pylori* infection and crowding, lifestyle, and hygiene factors. All crowding-related factors investigated, including family size, number of children, and sharing a bed, were significantly associated with being *H. pylori* positive (all *p* < 0.05). Interestingly, there was an increasing trend in percent feco-positive in both family size subgroups and number of children in household subgroups. The prevalence increased with the number of family members and with the number of children. Pupils who shared a bath towel or a washcloth with other members of household (OR = 1.49, 95% Cl: 1.03–2.17) were also significantly more affected with *H. pylori* infection, but no association was found for pupils who used a toothbrush shared with other members in the family (OR = 1.58, 95% Cl: 0.48–5.19). Prevalence of *H. pylori* was higher in children who rarely or sometimes washed their hands (91.5%) compared to those who always washed their hands (86.3%) with soap after go to toilet; OR = 1.71 (95% Cl: 1.13–2.59). The rate of infection was also higher in individuals who used only water (89.8%) or who used both water and paper (83.9%) compared to those who used only paper (74.2%) to clean after toilet; OR = 1.81 (95% Cl: 0.78–4.19) and OR = 3.06 (95% Cl: 1.33–7.04), respectively. The remaining hygiene factor (handwashing with soap before having meals) did not show association with *H. pylori* infection (*p* = 0.08). No significant differences were found between children who used or did not use bare hands to take food (*p* = 0.36). There was also no significant association between sharing cups or dishes or sauces or foods with other members in their and *H. pylori* detection (all *p* > 0.05).

When focusing on the two significant hygiene factors (methods used to wash hands and methods used to clean after toilet), the results showed that there was an increasing trend in prevalence of *H. pylori* infection: prevalence was the lowest in participants who always washed their hands with soap and used only paper to clean after toilet usage (303/372, 81.5%), followed by pupils who always washed their hands with soap but did not use paper to clean after toilet usage (608/684, 88.9%), and it was highest in individuals who rarely or sometimes washed hands with soap whatever using or not using paper to clean after toilet (323/353, 91.5%).

All water-related variables (mainwater source, drinking water source, cooking water source, and methods of water treatment) were not apparently associated with *H. pylori* infection (a *p* > 0.05) (Table 3).

**TABLE 3:** Association between *Helicobacter pylori* infection and selected factors in a multivariate analysis (Ho Chi Minh city, Vietnam. 2019).

Selected factors	n/N^a^	%	Unadjusted OR (95% Cl)	Adjusted OR (95% Cl)		* p*	
Age groups (years)^b^						0.002	
6–11	707/791	89.4	1.45 (1.06–1.99)	1.66 (1.18–2.32)			
≥12	527/618	85.3	1	1			
Gender^b^							
Female	614/716	85.8	1				
Male	620/693	89.5	2.66 (1.75–4.05)				
Living area^b^						<0.001	
Super-urban area	282/308	91.6	2.61 (1.58–4.31)	2.81 (1.67–4.72)			
Urban area	422/474	89.0	1.96 (1.29–2.96)	2.01 (1.29–3.09)			
Peri-urban area	310/354	87.6	1.69 (1.09–2.62)	1.72 (1.09–2.70)			
Rural area	220/273	80.6	1	1		
Family size						<0.001	
≤3	108/146	73.9	1	1			
4–6	848/960	88.3	2.66 (1.75–4.05)	3.04 (1.97–4.71)			
>6	278/303	91.8	3.91 (2.25–6.79)	4.09 (2.31–7.25)			
Number of children in household						
1	244/294	83.0	1				
2	773/872	88.7	1.60 (1.11–2.31)				
>2	217/243	89.3	1.71 (1.03–2.84)				
Sharing a bed							
Yes	981/1,104	88.9	1.64 (1.15–2.33)				
No	253/305	82.9	1				
Sharing bath towel or washcloth						
Yes	379/419	90.5	1.49 (1.03–2.17)				
No	855/990	86.4	1				
Handwashing with soap after toilet						0.02	
Always	911/1,056	86.3	1	1			
Rarely or sometimes	323/353	91.5	1.71 (1.13–2.59)	1.65 (1.08–2.55)			
Clean after toilet by using						0.004	
Water only	837/932	89.8	3.06 (1.33–7.04)	3.13 (1.28–7.64)			
Paper only	23/31	74.2	1	1			
Both water and paper	374/446	83.9	2.85 (1.29–6.43)	2.02 (0.82–5.01)			
Educational status of parents							
College/University	569/630	82.0	1				
High school	118/133	85.9	1.34 (0.85–2.13)				
Secondary school	440/512	88.7	1.73 (0.89–3.36)				
Primary school	141/172	90.3	2.05 (1.28–3.28)				

^a^ n/N: number of infected children/number of children within sub-group.

^b^ From this community-based study, a recent paper found that age, gender, and living area were associated with Helicobacter pylori infection [18].

In multivariate logistic regression analysis (Table 3), age, living area, size of family, methods used to wash hands, and ways to clean after toilet were significantly related to *H. pylori* infection. The prevalence of *H. pylori* was significantly higher in pulpils aged 9–11 years (OR = 1.66, 95% Cl: 1.18–2.32, *p* = 0.02) compared to those above 11 years old. It was also significantly increased with the level of crowded: peri-urban area (OR = 1.72, 95% Cl: 1.09–2.70, *p* < 0.001); urban area (OR = 2.01, 95% Cl: 1.29–3.09, *p* < 0.001); super-urban area (OR = 2.81, 95% Cl: 1.67–4.72, *p* < 0.001). Regarding to hygiene-related factors, *H. pylori* infection was significantly higher in pupils who rarely or sometimes handwash with soap after toilet (OR = 1.65, 95% Cl: 1.08–2.55, *p* = 0.02), and in children who used only water to clean after going to toilet (OR = 3.13, 95% Cl: 1.28–7.64, *p* = 0.004).

## Discussion

Overall, the prevalence of *H. pylori* infection among school-aged children in HCMC was 87.7%. Younger age, living in crowded areas (super-urban area, urban area, peri-urban area), larger family size, infrequency of handwashing with soap after toilet, using only water or both water and paper to clean after toilet were independently associated with an increased prevalence of paediatric *H. pylori* infection.

In our analysis, the prevalence of *H. pylori* significantly increased when the level of parent education decreased in univariate analysis, but this significant association was not observed in multiple analysis. This was explained by a correlation between level of education and living area. In fact, families with high level of education usually tend to live in central areas, where people can easily access most of commercial, educational, or business centers within a short distance. This inverse association was also comparable to a study of Nguyet et al. conducted in the North of Vietnam, which indicated low parental education as a risk factor of acquiring *H. pylori* [22]. This finding was also consistent with other reports from China [23], Pakistan [24], Iran [25] which also pointed that children whose parents had a lower education level were significantly at a higher risk of *H. pylori* infection. In addition, our study found that pupils with parents who completed only primary school had a higher prevalence than those with parents who completed a higher educational degree. A lower level of education can make harder to understand health message. A lower income level leads to live in smaller and more crowdy spaces, and so increased the chances to contract a *H. pylori* infection.

*H. pylori* infection was not significantly associated with the wealth status of a household, which was inconsistent with previous studies [26–28]. A possible reason for these conflicting findings might be the different methods used to assess the wealth status. Most previous studies used family monthly income and expenditure, or a combination of multiple indicators such as education level, occupation, and monthly earnings to categorize the wealth index. However, data on income and expenditure were not available in our study. We therefore built the household wealth index based on the ownership of 28 household assets following the DHS guidelines for Vietnam [21]. These items were easy to collect and to measure for researchers compared to using monetary indicators. However, the guideline was published several years ago and might be less appropriate at the time that our study was conducted. Therefore, further analyses are needed to discover and verify an appropriate method for evaluating the wealth index of Vietnamese population.

*H. pylori* could spread from person to person through two main routes: the oral-to-oral route, and the fecal-oral route. The second route was identified to be the predominant mode for *H. pylori* transmission in several studies [10, 29]. In addition, the pathogen in feces can spread by the fecal-oral route from one person to another’s oral cavity through contaminated food, surfaces, or water, which is mainly caused by a lack of adequate sanitation and poor hygiene practices [3, 9, 29]. In our findings, the prevalence of *H. pylori* infection was significantly higher in pupils who infrequently washed their hands with soap after toilet, and it was significantly lower in participants who used only paper to clean after toilet. Interestingly, when carefully assessing the two hygiene factors, results showed that there was an increasing trend in prevalence of *H. pylori* infection: lowest in participants who always washed their hands with soap and used only paper to clean after toilet usage (81.5%), followed by pupils who always washed their hands with soap and did not use paper to clean after toilet usage (88.9%), and highest in individuals who rarely or sometimes washed hands with soap whatever using or not using paper to clean after toilet (91.5%). These evidences support that unhygienic toilet conditions, particularly poor handwashing behavior, increase the possibility of contact with fecal material, which can create an infection vehicle of *H. pylori* and cause the spread of the bacterium both within the family and within the community. In addition, sharing cups or dishes or sauces or foods was not associated with *H. pylori* infection in our findings, suggesting that the oral-oral transmission might not play a main route in spreading *H. pylori* in children in HCMC. Based on these findings, the fecal-oral route appears to be the most likely mode of transmission for *H. pylori* infection among school-aged children in HCMC. Therefore, educating and improving hygiene practices are essential to control the transmission of *H. pylori* and reduce the burden of this infection in the community.

Waterborne infection can be an important route of *H. pylori* infection, particularly in developing countries with a high prevalence of acquiring *H. pylori* [9]. Several studies pointed to a positive association between *H. pylori* infection and the consumption of well water [10, 30]. Other reports also found a strong correlation between *H. pylori* infection and the source of drinking water [27, 31]. However, water-related variables (source of drinking water or cooking water or methods of water treatment) were not correlated with *H. pylori* prevalence in our study, supporting that the water supply may not act as a reservoir for the transmission of *H. pylori* in the population of HCMC. Indeed, HCMC replaced, developed, and installed new water distribution systems over the past 10 years to ensure treated water for 100% population of the city from 2015 [32]. However, some households are still using well water instead of treated water from the government.

Our study is the first to use a random, so representative sample of school-aged children of HCMC to investigate the pathway of *H. pylori* transmission. Our results point the oral-fecal route as an important - but not isolated - pathway for *H. pylori* spread. Other routes of transmission such as oral-oral are not well documented yet.

Our study has several limitations. Firstly, we relied on the DHS guidelines to build a wealth index based on 28 household assets, but this approach may not be the most suitable way for assessing the wealth status of the family. Several studies showed that a combination of multiple indicators, including education level, occupation, and monetary indicators such as monthly income and expenditure, provides a more comprehensive wealth index compared to solely replying on house assets [26–28]. However, our study lacked data on monthly income and expenditure, which limited our ability to use those measures to evaluate the wealth status. Secondly, we did not examine the school environmental fators as availability of soap and water at handwashing sites, food-borne factors, sleeping conditions, that could potentially have a significant impact on the prevalence of *H. pylori* infection.

### Conclusion

The prevalence of *H. pylori* among school-aged children of HCMC remains high. Younger age, crowded living areas, larger family size, and poor hygienic practices were found to be positively associated with a higher prevalence of *H. pylori* infection among school-aged children of HCMC. These findings highlight the importance of the fecal-oral route and the attribution of crowded living conditions to the spreading of *H. pylori* in HCMC. To decrease the acquisition of *H. pylori* as well as the burden of *H. pylori* among pupils in HCMC, efficient preventive programs should be set up with a focus on education of hygiene practices, and oriented to those living in crowded conditions. Further analyses are needed to address the impact of school environment on *H. pylori* transmission.
