# Supplementary material for: Factors Associated With Helicobacter Pylori Infection Among School-Aged Children From a High Prevalence Area in Vietnam
Source: Int J Public Health. 2023 May 11;68:1605908. doi: 10.3389/ijph.2023.1605908 (PMC10209423; doi:10.3389/ijph.2023.1605908)
Supplement: Supplementary file 1 [file Table1.DOCX]

| **No** | **District name** | **n** | **N** |
| --- | --- | --- | --- |
| 1 | District 1 | 81 | 59,883 |
| 2 | District 2 | 67 | 49,624 |
| 3 | District 3 | 52 | 38,678 |
| 4 | District 4 | 21 | 20,675 |
| 5 | District 5 | 76 | 56,618 |
| 6 | District 6 | 68 | 52,038 |
| 7 | District 7 | 34 | 24,732 |
| 8 | District 8 | 45 | 32,276 |
| 9 | District 9 | 114 | 82,192 |
| 10 | District 10 | 43 | 31,376 |
| 11 | District 11 | 45 | 33,107 |
| 12 | District 12 | 47 | 30,693 |
| 13 | BINH THANH | 79 | 57,859 |
| 14 | GO VAP | 53 | 40,364 |
| 15 | PHU NHUAN | 78 | 56,385 |
| 16 | TAN BINH | 81 | 60,108 |
| 17 | TAN PHU | 93 | 67,842 |
| 18 | BINH TAN | 69 | 49,964 |
| 19 | THU DUC | 33 | 24,192 |
| 20 | BINH CHANH | 57 | 41,837 |
| 21 | CU CHI | 78 | 56,014 |
| 22 | CAN GIO | 27 | 18,139 |
| 23 | HOCMON | 84 | 59,275 |
| 24 | NHA BE | 51 | 33,234 |
| *N: total number of pupils in the district n: number of children participating in our study* | | | |

I**nternational Journal of Public Health**

**Figure 3.** Mapping of the number of 6 – 15 years children selected in each of the 24 districts of Ho Chi Minh city (Ho Chi Minh city, Vietnam. 2019)


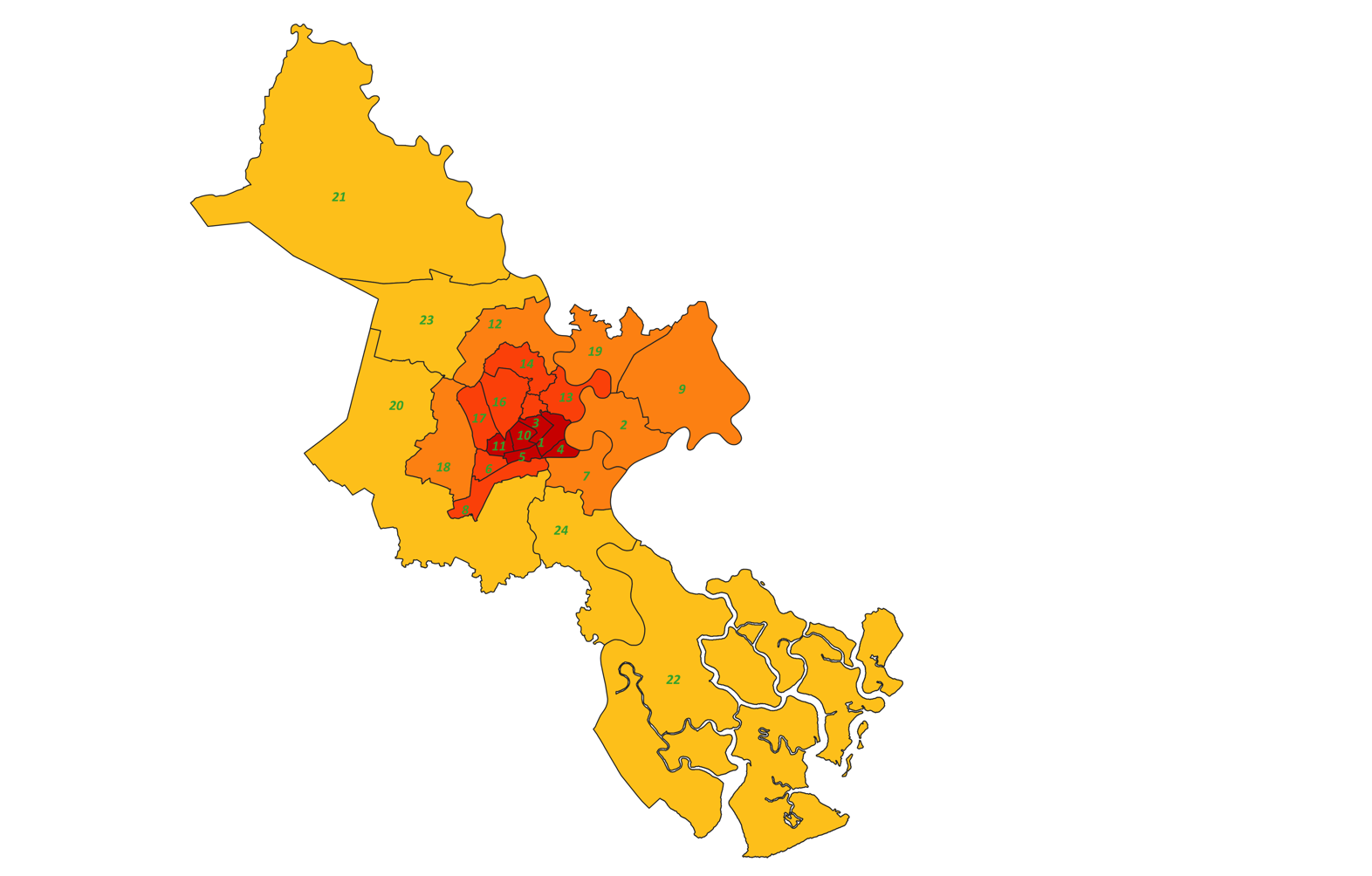


Super-urban area

Urban area

Peri-urban area

Rural area

**Factors associated with Helicobacter Pylori Infection Among School-aged Children From a High Prevalence Area in Vietnam**
